# On-Line Corrosion Monitoring of Plate Structures Based on Guided Wave Tomography Using Piezoelectric Sensors

**DOI:** 10.3390/s17122882

**Published:** 2017-12-12

**Authors:** Jing Rao, Madis Ratassepp, Danylo Lisevych, Mahadhir Hamzah Caffoor, Zheng Fan

**Affiliations:** 1 School of Mechanical & Aerospace Engineering, Nanyang Technological University, 50 Nanyang Avenue, Singapore 639798, Singapore; jrao001@e.ntu.edu.sg (J.R.); madis.ratassepp@ttu.ee (M.R.); Dani.Lisevych@ntu.edu.sg (D.L.); mahadhir.hamzah@lr.org (M.H.C.); 2 Department of Civil Engineering and Architecture, Tallinn University of Technology, Ehitajate tee 5, 19086 Tallinn, Estonia

**Keywords:** corrosion monitoring, guided wave tomography, full waveform inversion, piezoelectric transducers

## Abstract

Corrosion is a major safety and economic concern to various industries. In this paper, a novel ultrasonic guided wave tomography (GWT) system based on self-designed piezoelectric sensors is presented for on-line corrosion monitoring of large plate-like structures. Accurate thickness reconstruction of corrosion damages is achieved by using the dispersive regimes of selected guided waves and a reconstruction algorithm based on full waveform inversion (FWI). The system makes use of an array of miniaturised piezoelectric transducers that are capable of exciting and receiving highly dispersive A0 Lamb wave mode at low frequencies. The scattering from transducer array has been found to have a small effect on the thickness reconstruction. The efficiency and the accuracy of the new system have been demonstrated through continuous forced corrosion experiments. The FWI reconstructed thicknesses show good agreement with analytical predictions obtained by Faraday’s law and laser measurements, and more importantly, the thickness images closely resemble the actual corrosion sites.

## 1. Introduction

Corrosion is a dynamic process which can be influenced by many factors including time and process variables. Therefore, the monitoring of corrosion processes has become highly sought-after to enhance the safety and the profitability of petrochemical plants. The relatively long time intervals associated with off-line inspections [1] render these techniques incapable of revealing unforeseeable changes which could have developed during corrosion progression. On-line electrochemical approaches such as linear polarization resistance measurement [2,3] and weight measurement [3,4] are intrusive and delicate as they require contact with the corrosive substances. Ultrasonic techniques, on the other hand, provide a non-intrusive means for the on-line monitoring of corrosion [5,6].

A small number of permanently installed point thickness gauges has been used for monitoring the average thickness loss over a modest area [7,8], which gives high probability of detection. However, if the requirement is to detect relatively severe and highly localized defects, the number of point sensors required substantially increases. In these cases, the area monitoring systems such as the field signature method [9] or dispersive guided wave velocity measurements [10,11] appear to be more attractive. Nonetheless, the area monitoring systems are only sensitive to the average loss of the wall thickness over a monitored area. If a wall loss is not uniform, these systems would either not be able to detect it or underestimate the severity of corrosion as the average thickness loss is taken as representative. In cases of severe wall loss over a large area coverage, guided wave tomography (GWT) system has demonstrated the capability and sensitivity of mapping non-uniform, localized corrosion damages [12,13,14,15]. It reconstructs the velocity map by the inversion of signals measured by an array of transducers around the perimeter of the inspection area, which can be converted to the thickness map by the dispersion relations of selected guided modes. Therefore, GWT can achieve accurate sizing of corrosion defects in the inspected area within the array.

Generally speaking, a GWT system consists of an array of sensors, a data acquisition system, data processing techniques and a reconstruction algorithm. A successful GWT system ought to have good sensitivity to corrosion damages, localization and imaging capabilities, with a low transducer density [16]. Ultrasonic non-destructive evaluation systems based on the guided waves generated by arrays of electromagnetic acoustic transducers (EMATs) have been applied to the corrosion monitoring of pipes and plate-like structures [17]. For example, the received signals from arrays of EMATs encircled the pipe were processed by using ultrasonic computerized tomography [18,19] to generate the maps of thickness loss. However, EMATs have a relatively low signal-to-noise ratio (SNR) and their performance relies on the electromagnetic properties of the tested objects such as the presence of a highly magnetostrictive oxide layer [20]. By contrast, piezoelectric transducers have high SNR, relatively low cost and small energy consumption which are more appropriate for long-term monitoring [21,22,23,24,25]. However, it has previously been shown that the scattering from transducers can affect the performance of the thickness reconstruction and it needs to be minimized for more accurate reconstruction [12]. In this study, an optimized low frequency A0 mode piezoelectric transducer is designed for the GWT system and the effect of transducer array scattering on the accuracy of the thickness reconstruction is also investigated.

In this paper, the system consists of self-designed piezoelectric transducer arrays, automated signal generation, acquisition and processing, and a reconstruction algorithm using a full waveform inversion (FWI) approach. As such, the automated on-line monitoring of corrosion progression can be done efficiently and with little manual labor. The performances of the system are examined in a lab-based continuous forced corrosion experiments.

In the following section, a GWT system is described, including the array system, data acquisition, FWI algorithm and data processing method. In Section 3, the scattering from transducers and its effect on the thickness reconstruction are investigated. In Section 4, forced corrosion and thickness measurements using laser profilometry are presented. The results from experiments and discussions are followed in Section 5. Conclusions are summarized in Section 6.

## 2. Monitoring System

### 2.1. Array System and Experimental Setup

**Figure 1:**
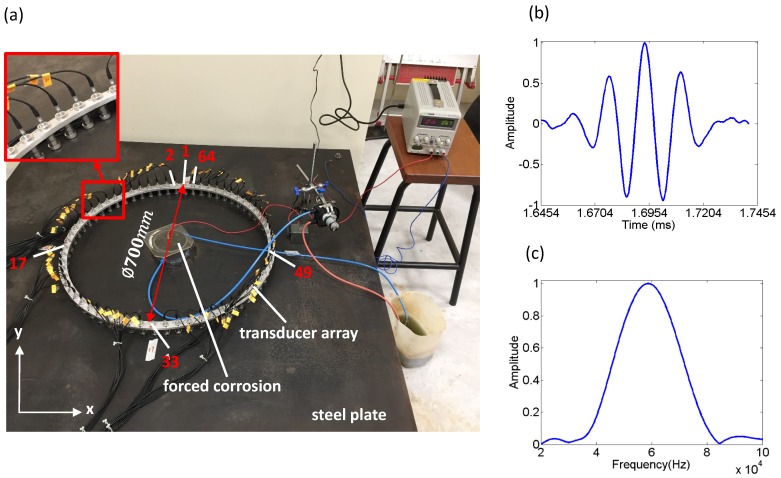
(**a**) Setup of the guided wave tomography measurements. All experiments were carried out at room temperature. (**b**) Time trace and (**c**) frequency spectrum of a received signal from the experiment without defects in the steel plate. The source was at point 1 and the measurement was taken at point 17.

An array consisting of 64 piezoelectric stack transducers was used in the experiment, which were uniformly distributed on a circular array of diameter of 700 mm, as shown in Figure 1a. The transducer was designed to generate the A0 guided wave between 30 kHz to 90 kHz. The design was based on the stacking of piezoelectric elements together with a front layer to maximize the generation and receiving efficiency of the selected guided mode and meanwhile minimize the dimensions of the transducer [26]. Piezoelectric ceramic PZT-5A element with 10 mm in diameter and 2 mm in thickness was used. The waveform generator was controlled by an ultrasonic phased array controller (Lecoeur Electronique, Chuelles, France). A 5-cycle Hanning windowed tone-burst signal with the central frequency of 60 kHz was selected for the excitation. Figure 1b,c show a typical time trace of a received A0 mode, excited at point 1 and measured at point 17 (distance = 350$\sqrt{2}$ mm) in a 10 mm thick steel plate and its frequency spectrum. The 64 transducers were held in place by a support ring to ensure the accuracy in their positioning in Figure 1a. In order to ensure good contact between the plate and each of the transducers, couplant was used and then weights were evenly distributed on the mounting bracket of the support ring, with a spring fitted between the support ring and each individual transducer. In the experiments, only transmitted signals from the transmission subset were used here to avoid the reflection from the edge of the plate [27]. Using the current system, SNR greater than 30 dB can be attained after five averages.

A 1500 mm × 1500 mm × 10 mm mild steel plate was selected for the experiment, and a corrosion site was designed in the center of the plate, as shown in Figure 1a. It is worth mentioning that prior to exposing the steel plate to corrosion, an artificial defect with the diameter of 80 mm and depth of 1 mm was milled in the center of the plate. Such approach will lead the subsequent corrosion to a smooth transition rather than a step change in depth from the top of the plate to the bottom of the defect. Less mode conversions (the A0 to S0 mode) are expected from relatively smooth thickness changes, favoring the acoustic assumption of the thickness reconstruction algorithm [28].

### 2.2. Imaging Algorithm

**Figure 2:**
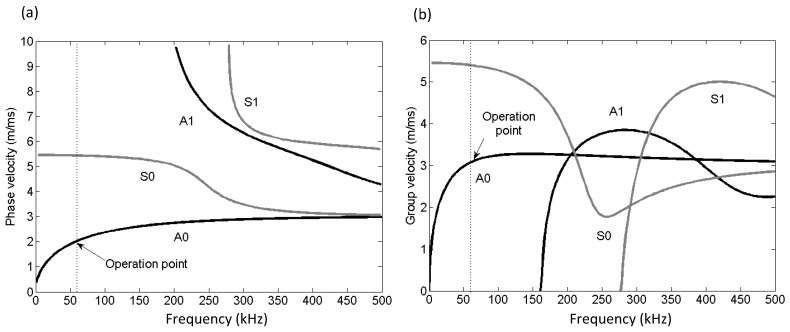
Dispersion curves of Lamb waves in a 10 mm thick steel plate. (**a**) Phase velocity and (**b**) group velocity.

In this work, the GWT uses the assumption that the guided wave propagating in the plate of varying thickness behaves the same as the acoustic wave propagating in 2D medium with varying velocity [27,28]. For the acoustic assumption, the dispersion relationship is exploited through converting the velocity to the thickness at a particular frequency, as shown in Figure 2.

In this paper, the thickness reconstructions from simulation data and real inspection data collected from a piezoelectric stack transducer array are achieved by the FWI algorithm [27]. It uses a forward solver to predict the scattering of guided wave through corrosion defects, and an iterative inversion model to reconstruct the corrosion profile. At each iteration, numerical modeling is carried out with the aim of least-squared minimization of the residual data between the predicted data by the model and the observed data from simulations or experiments. This algorithm overcomes the limitation of linear scattering, and higher order diffraction and scattering can be taken into account in its numerical solver. Thus, it is possible to achieve accurate inversion results.

A forward model is developed to solve frequency domain 2D acoustic wave equation by the Finite Difference (FD) method, which can be written in matrix form

(1) $$\begin{array}{c} AP=S, \end{array}$$

where A is the forward modeling operator (i.e., complex-valued impedance matrix). The pressure wavefield P and the source term S are stored as vectors of dimension $n_{x}\times n_{y}$, where $n_{x}$ and $n_{y}$ represent the dimensions of the regular FD grid. When the matrix A was factorized by LU decomposition, the solutions for multiple sources can be efficiently obtained by forward and backward substitutions. Therefore, Equation (1) can be given by

(2) $$\begin{array}{c} \mathrm{LU}\left[P_{1}P_{2}\cdots P_{n}\right]=\left[S_{1}S_{2}\cdots S_{n}\right], \end{array}$$

where n is the number of the sources. This makes multiple-source problems easier to be solved in the frequency domain [29,30]. The wave equation is discretized in a FD sense by using the mixed-grid approach [31,32,33].

The inversion is to minimize the objective function, which can be solved by the gradient method. The gradient *G* can be obtained by zero-lag convolution of the forward wavefield with the backward propagated residual wavefield based on the reciprocity principle [34]. It can be expressed as

(3) G(k)=Re{JtWdΔd*},

where Re is the real part of a complex number and *k* is the iteration number. $J^{t}$ is the transpose of the Jacobian matrix and $W_{d}$ is a weighting operator. $\Delta d^{*}$ denotes the conjugate of the data residual between the data computed in model m and the observed data. In order to obtain reliable results, the scaling and regularization should be applied in the gradient, and more details can be found in [33,35]. Then, a model parameter vector m is updated iteratively using the steepest descent algorithm as follows:

(4) $$\begin{array}{c} m^{\left(k+1\right)}=m^{\left(k\right)}-\alpha G^{\left(k\right)}. \end{array}$$

where α is the scalar step length estimated by parabolic fitting [36].

In this work, the inversion is started from lower frequencies where the global minimum can be more easily found as the velocity errors in the waveforms remain below a half cycle [37,38]. After inverting for the larger scaled model structures in lower frequencies, higher frequencies are then used to obtain the finer model. Therefore, this multiple-scale strategy can help to mitigate the non-linearity of the inverse problems and reach the global minimum.

### 2.3. Data Processing and Inversion

The experimental signals need to be calibrated before they can be used for the inversion. The calibration is to fit the measured data from the experiment with the FD modeling data used in the forward model. In this paper, the calibration factor is determined by calculating the ratio of the required frequency domain data of the homogeneous background model (without defects) by the FD method and the experiment on the homogeneous plate, similarly as in [27].

At each FWI run, the homogeneous velocity distribution at the lowest frequency is used as the starting model and the sequential inversion with increasing frequencies is applied. Since global minima can be found more easily at lower frequency where the velocity model resembles to the homogeneous background model [38,39]. In this study, sequential frequencies of 35, 46 and 60 kHz are used. The inversion is regularized by the Gaussian spatial filter of the perturbation model to minimize high-frequency artifacts in the gradient. After the inversion, the reconstructed velocity map is converted to the thickness map by the dispersion relationship.

## 3. Scattering from Transducers and Its Effect on the Thickness Reconstruction

### 3.1. Scattering from Transducers

In the imaging algorithm, the forward modeling is based on the assumption that the incident wavefield is generated by a point excitation transducer and the scattering from the defect is produced by this incident wavefield. However, in the case of contact transducers array, the incident wave can interact with other transducers, causing secondary incident wavefields that can produce unwanted scattering components from the defect. This can affect the reconstruction of the defect [12]. Therefore, it is important to evaluate the scattering from transducers and its impact on the thickness reconstruction.

**Figure 3:**
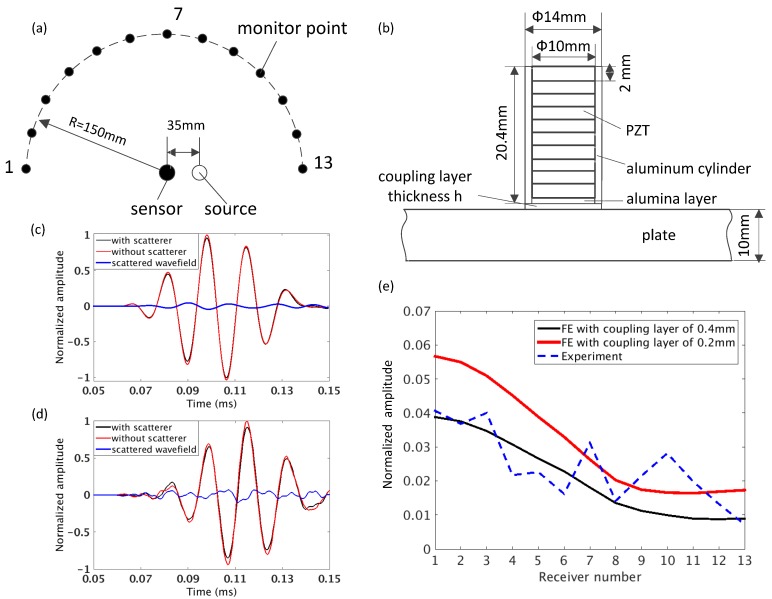
(**a**) Schematic of measurement for the scattering transducer in the center of the array by using the symmetry in this case with one source and 13 monitor points; (**b**) cross section of modeled transducer, the scattering transducer, in finite element (FE) model; (**c**) scattered wavefield obtained by subtracting the incident wavefield of the FE simulation with and without the scatterer in measured point 1; (**d**) scattered wavefield obtained from the experiment in measured point 1 and (**e**) the normalized amplitude varying with the measured points in the simulation and the experiment.

Finite element (FE) simulations carried out by using software Abaqus and experiments were used to investigate the scattering from a single transducer attached to the steel plate. The wave generated from a transducer interacting with its neighbor in the designed array was modeled. The setup is shown in Figure 3a. The scattering transducer is located in the center of a 300 mm diameter array and the source transducer is located 35 mm away from the center.

The cross section of the scattering transducer attached to the steel plate used in the FE model is shown in Figure 3b and the properties of materials are given in Table 1. The transducer is composed of an aluminum cylinder, a 0.4 mm thick alumina front layer and a 20 mm thick stack of 10-PZT discs. The contact between the transducer and the plate was modeled by a thin coupling layer (density = 1100 kg/m^3^ and acoustic impedance = 1.84 × 10^6^ kg/m^2^ s). The model was meshed with cubic eight node elements with the size of 1 mm, and smaller elements for the coupling and the front layer. The A0 mode was excited by an out-of-plane force at the plate surface and out-of-plane displacements were measured at 13 monitor points equally spaced along a half circular array, as shown in Figure 3a. The excitation signal was a 5-cycle Hanning windowed tone-burst at the central frequency of 60 kHz, the same as the one used in the thickness reconstruction. The scattered wavefield was obtained by subtracting the incident wavefield of the model with and without the scatterer, as shown in Figure 3c.

The experiments were performed on the steel plate described in Section 2.1 and two transducers from the array were used for the measurements. The scattering transducer was attached to the plate by adding weight on it. The A0 mode was excited by using Tiepie Handyscope HS3 and the out-of-plane displacements were measured by a Polytec OFV-505 laser vibrometer. The scattered wavefield was extracted in the same manner as in FE modeling, as shown in Figure 3d.

Figure 3e shows the amplitude of the scattered wavefield from the transducer at 60 kHz with respect to different monitoring positions. It is normalized by the amplitude of the incident wave with the same propagation distance. Since the exact thickness of the coupling layer in the experiment is unknown, two reasonable assumptions of 0.2 mm and 0.4 mm were used in the FE model for comparison. It can be seen that overall the scattered wavefield is weak, with the amplitude decreasing from the transmission part (closer to monitor point 1) to the reflection part (closer to monitor point 13). It can also be noted from the figure that thinner coupling layer results in stronger scattering. The reason is that in the case of thinner layer, more energy from the wave propagating in the plate is transmitted to the couplant [40] and thus more energy is converted into the scattering. It can be seen that the trend shown in the experimental results agrees well with the predictions from the FE simulation, despite some variations due to the noise.

### 3.2. Scattering Effect on the Reconstruction

The transducer scattering effect on the thickness reconstruction was investigated by FE modeling. A Hann-shaped defect with the surface diameter of 120 mm and the depth of 3 mm was modeled at the center of a steel plate with the dimension of 1100 mm × 1100 mm × 10 mm, the same as the one shown in our previous work [41]. A 700 mm diameter array with 64 transducers was simulated. Two cases were considered: the array with artificial transducers (waves generated on the nodal position) and the one with real transducers introduced in Section 3.1. The same input signal and reconstruction method were applied in the simulation.

**Figure 4:**
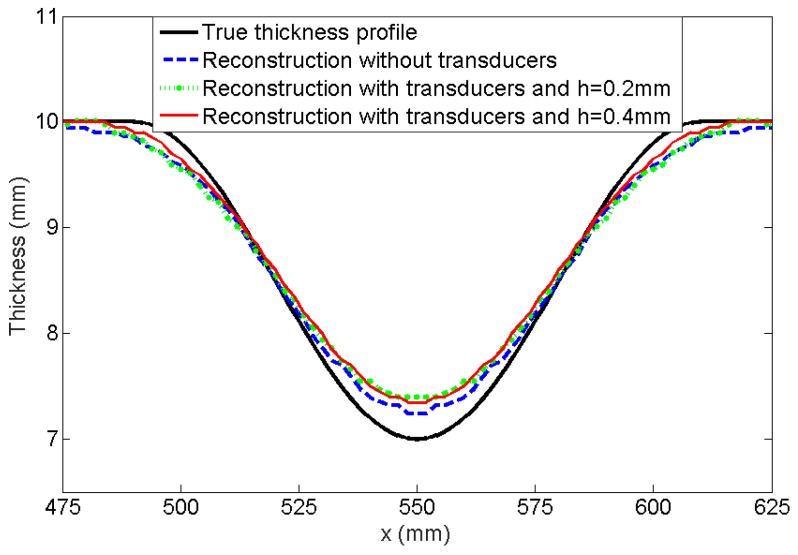
Thickness profiles across the reconstructions of a Hann-shaped defect with the surface diameter of 120 mm and the depth of 3 mm from the FE model without and with an array of modeled transducers. *h* is the thickness of the coupling layer.

The cross sections of the reconstructed thickness map are presented in Figure 4. Two coupling layers of 0.2 mm and 0.4 mm thick were also modeled. It can be seen that the reconstructed thickness profiles obtained from artificial and real transducers are almost superposed, indicating that the scattering from the transducers has minimal effect on the thickness reconstruction.

## 4. Experimental Simulation of Corrosion Damage

### 4.1. Forced Electrochemical Corrosion

**Figure 5:**
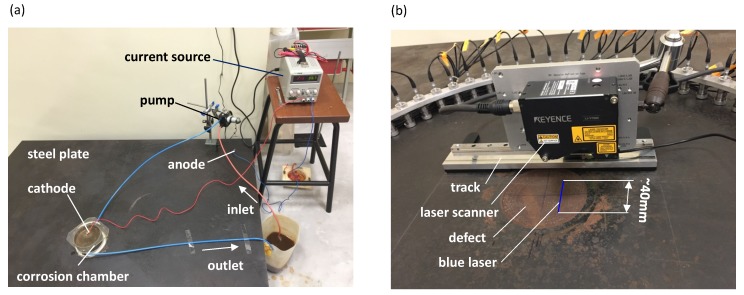
(**a**) The experimental setup of the forced corrosion and (**b**) a laser profile scan of the corrosion defect in a 10 mm steel plate.

While the unforced corrosion is more representative of real-world cases, the forced corrosion, which is driven by an applied electric current, is used for accelerating corrosion damage for the purposes of obtaining results in a relatively short time [42]. For the corrosion of steel, this reaction is dominated by the two-electron oxidation of elemental iron, $Fe\to Fe^{2+}+2e^{-}$. In the forced corrosion experiments carried out in this work, the mild steel plate was made the anode on which the oxidation of element iron occurs, and a steel thin disk inserted into the chamber (shown in Figure 5a) acts as the cathode.

Figure 5a shows the experimental setup of forced electrochemical corrosion on the mild steel plate. The corrosion chamber with a diameter of 130 mm is attached to the surface of the steel plate, which is the concentric location of the artificial defect. In the corrosion process, a brine electrolyte is circulated through the chamber by a pump (Verderflex M025, Verder Ltd., Castleford, UK) in order to prevent the precipitation of corrosion product iron oxide. This will help to maintain the rate of corrosion and to prevent the further oxidation of iron oxide into iron hydroxide which is insoluble and rather protective against further corrosion. Also, it can minimize the temperature change during the electrochemical corrosion process because the obvious increase in temperature causes the reductions in ultrasonic velocities [43]. In the forced corrosion experiment, the electric current is supplied by a constant current source (IPS 303DD, ISO TECH, Southport, UK).

Faraday’s law of electrolysis is used to predict the average wall loss due to an applied current in the forced corrosion experiment. According to this law, the average wall loss $\Delta T_{h}$ of the steel plate can be calculated by

(5) $$\begin{array}{c} \Delta T_{h}=-\frac{M_{Fe}It}{2FA_{c}\rho_{Fe}}, \end{array}$$

where $M_{Fe}$ is molar mass of steel (0.055845 mol/kg), *I* being applied current, and *t* is the elapsed time. *F* is the Faraday’s constant (96,485.3329), $A_{c}$ being the area of the corrosion surface (diameter of 0.13 m) and $\rho_{Fe}$ is the density of steel plate (7850 $\mathrm{kg}/m^{3}$).

In this study, the constant current of 3 A was used and thus the relationship between the wall loss of steel plate and time is given by

(6) $$\begin{array}{c} \Delta T_{h}=-8.3324\times 10^{-9}t. \end{array}$$

### 4.2. Thickness Measurement Using Laser Profilometry

In this work, a blue laser scanner (LJ-V7080, KEYENCE, Osaka, Japan) was employed to measure the wall losses and profiles of the controlled corrosion damage, as shown in Figure 5b. It has the repeatability precision of 10 μm along the width of the laser beam, and 0.5 μm along the thickness of the plate. The results are post-processed by using the software supplied by the manufacturer. It needs to be noted that the largest measuring range of laser in width is around 40 mm and thus multiple measurements should be taken and merged together for a larger size of the corrosion damage.

Before measuring the corrosion profile of the plate with the laser scanner, the forced corrosion setup is removed and the residual corrosion products on the corrosion surface are carefully cleaned away.

## 5. Results and Discussion

The focus of this paper lies on the performance of the GWT system for continuous, on-line corrosion monitoring. Firstly, the reconstructed depths of the corrosion defect at different stages using the FWI algorithm are compared with the values predicted by Faraday’s law. This is followed by a detailed comparison of thickness maps, obtained by the FWI algorithm and laser profilometry, at two different corrosion periods.

### 5.1. Wall Loss Monitoring

In this work, measurements of the corrosion damage induced by forced corrosion were carried out over a period of approximately 110 h. According to Equation (6), the average thickness loss of 1 mm can be achieved around 36 h.

To compare the average reconstruction by FWI with the average wall loss calculated by Faraday’s law, an alternative metric for quantifying the average reconstructed thickness from FWI is to take the mean value of all reconstructed thickness within the upper limit of the minimum reconstructed thickness (1+5%)$T_{min}$ [28]. The average reconstructed thickness $\bar{T}$ can be given by

(7) $$\begin{array}{c} \begin{array}{c} \bar{T}=\sum_{n=1}^{N}\frac{T(x_{n},y_{n})}{N}\;when\;T\left(x_{n},y_{n}\right)\leq \left(1+5\%\right)\times T_{min}, \end{array} \end{array}$$

where $T(x_{n},y_{n})$ is the reconstructed thickness at coordinate $x_{n}$ and $y_{n}$; $T_{min}$ is the minimum reconstructed thickness and *N* is the reconstructed number according to the range within $\left(1+5\%\right)T_{min}$.

**Figure 6:**
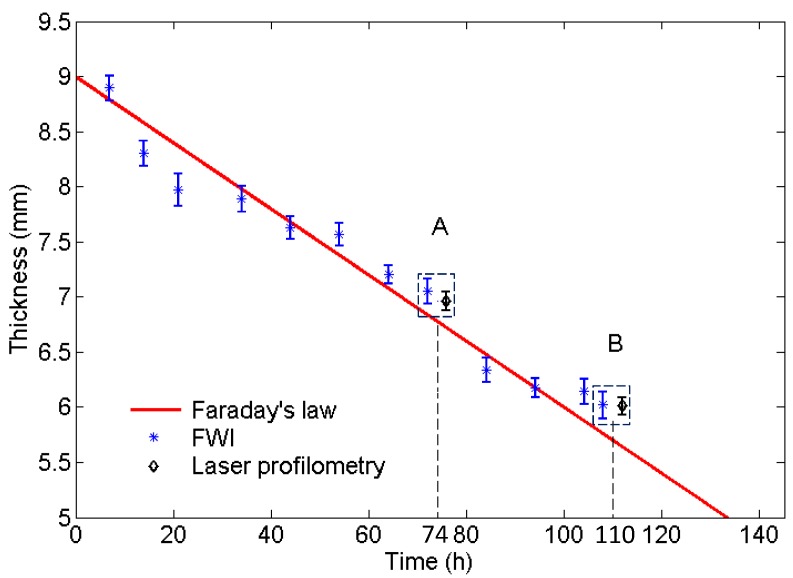
The comparison of the average reconstructed thickness of the defect obtained by full waveform inversion (FWI) at 60 kHz and predictions by Faraday’s law in different corrosion periods of a mild steel plate. The upper and lower limits of measured thickness of defect A and B at corrosion time of 74 and 110 h obtained by FWI and laser profilometry are shown in the rectangular boxes, respectively. The results of laser profilometry will be explained in Section 5.2.

Figure 6 shows the average reconstructed thicknesses of defects at different stages during the corrosion process by the FWI algorithm, together with the analytical predictions by Faraday’s law. The wall losses are presented with error bars, showing the upper and lower limit of the measured thickness. It can be observed that all average reconstructed thicknesses are in excellent agreement with the analytical predictions in the continuous corrosion.

### 5.2. Thickness Reconstruction

**Figure 7:**
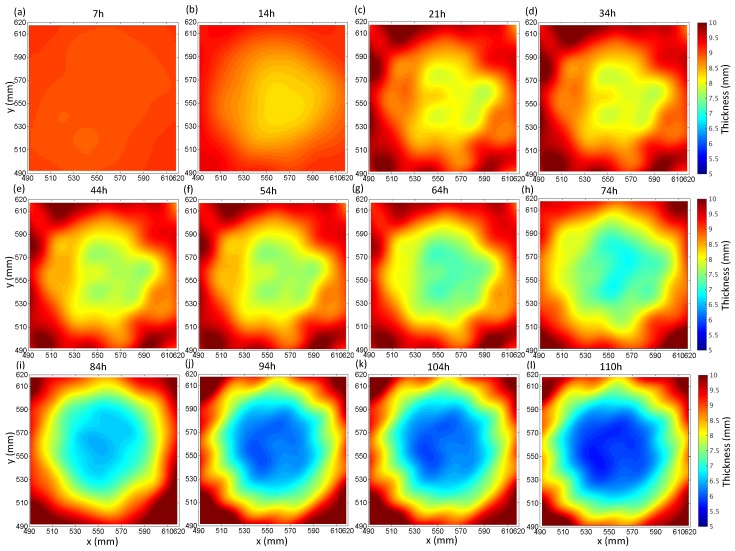
Thickness mapping of the continuous corrosion defect by FWI at 60 kHz. (**a**–**l**) reconstructed thickness in different corrosion periods corresponding to Figure 6.

In [41], it has been shown that the FWI algorithm achieves a resolution around 1.5–2 wavelengths for the majority of defect depths from the experiment. Also, the shape of the defect profile has an influence on the resolvability. Here, the verification of the effect on reconstruction accuracy is carried out by different surface and bottom diameters, and the depths of flat-bottom defects in different corrosion progression. Figure 7 demonstrates the thickness mapping obtained by the FWI algorithm at different corrosion time stages shown in Figure 6. The results of corrosion defect A and B at the corrosion time of 74 and 110 h based on the GWT and a laser profile scan in two dotted boxes are shown in Figure 6. Excellent agreements are seen between the reconstructions from the GWT and the laser profile measurements, even closer than the predictions from the analytical model.

**Figure 8:**
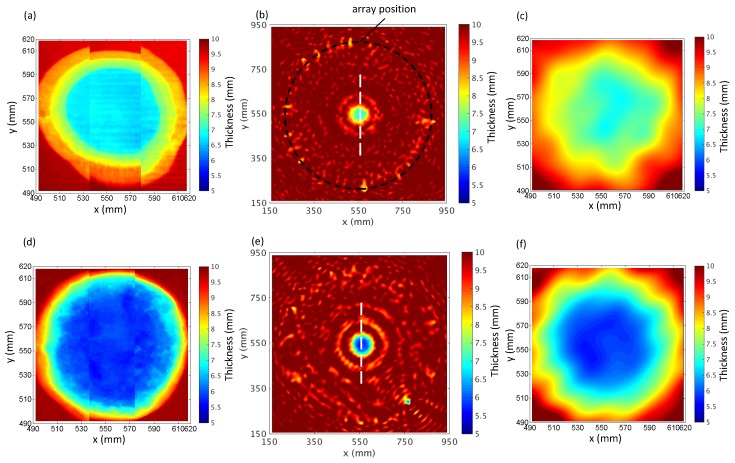
Thickness mapping of the corrosion defect A and B. (**a**) Laser measured profile of defect A after post-processing, (**b**) thickness reconstruction from FWI at 60 kHz and (**c**) a zoomed-in view of defect A from (**b**). The dashed curve presents the position of the array. (**d**) Laser measured profile of defect B after post-processing, (**e**) thickness reconstruction from FWI at 60 kHz and (**f**) a zoomed picture of defect B from (**e**).

Figure 8a shows the profile measured by laser scans after 74 h of corrosion. As mentioned earlier, due to the limitation of the laser scanner, the profile of the defect needs to be scanned by separate strips, and therefore the profile spliced from the scans is not perfectly smooth. However, it is clear from the figure that the quality of the interior of the resolvable area has not been degraded. The defect has a regular shape and relatively smooth variations in the thickness. The largest extent is around 130 mm (≃3.9 wavelengths at 60 kHz) and the deepest depth is about 3 mm. Figure 8b,c present the thickness reconstruction results and the zoomed image of this defect. It can be seen that the overall shape of defect A reconstruction is well approximated, when compared with the measured corrosion profile. However, the experimental reconstruction has slight deformation in the shape of the defect and the contrast of the defect is also slightly underestimated. The reason is that the reconstructed thickness is affected by the effect of different scattering behavior between guided waves with thickness variation and acoustic waves with velocity variation [44]. Some artifacts can be observed in the reconstructed image, especially at the positions of the transducers array and around the defect. There could be a few sources for the artifacts. Besides the environmental noise in the experiment, artifacts at the array position can be affected by the undersampling of the wavefield [45] and the scattering from the array of transducers. The artifacts around the defects could mainly be produced by the attached chamber wall and silicon used in the bond of the chamber wall and the plate, which have not been taken into account in the reconstruction algorithm.

The thickness profiles of a deeper defect B after 110 h of corrosion are shown in Figure 8d,f. It is clear that the reconstruction can still capture most features of the defect, although the discrepancy compared with the measured thickness profile is larger than the previous case, as the error of the reconstruction increases with the depth of the defect [19].

**Figure 9:**
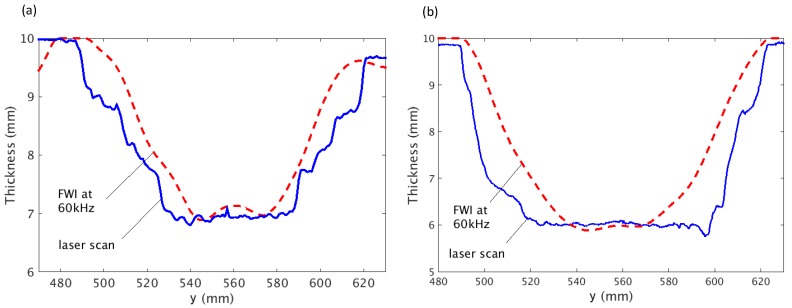
Cross sections of FWI reconstruction and laser measurement of (**a**) defect A and (**b**) defect B along the vertical line.

The cross sections of the thickness profile along the vertical direction (dashed lines in Figure 8) for both defects are plotted in Figure 9. It can be seen that location of the defect and the minimum thickness have been successfully identified for both cases. It can also be noted that the reconstructed defects are slightly narrower than the measurement from the laser scanner, which is believed to be caused by the difference in the scattering by the acoustic and the elastic model [44].

## 6. Conclusions

In this paper, a GWT system for the efficient and accurate on-line corrosion monitoring of plate-like structures has been presented. A circular array of piezoelectric transducers has been designed to generate and receive the A0 Lamb wave at low frequencies. It was found that the influence of the scattering from the selected transducers on the accuracy of the thickness reconstruction is minimum. In the forced corrosion experiment, the average wall losses that were reconstructed by FWI agreed well with the analytical predictions obtained by Faraday’s law. The reconstructed thickness maps at two different stages matched well with the measurements from a laser scanner. Therefore, the GWT system presented in this paper could be a powerful tool for the corrosion monitoring in both the laboratory and the field. As part of the future work, the thermal stability of GWT for the on-line and real-time corrosion monitoring of the wall loss in pipes will be investigated.

## Figures and Tables

**Table 1:** Material properties.

Material	Young’s Modulus (GPa)	Poisson’s Ratio	Density (kg/m^3^)
Steel	210	0.287	7850
Aluminum	70	0.33	2700
Alumina	300	0.21	3860
PZT	50	0.35	7750
